# [Expression of Concern] TGF-β1 mediates epithelial to mesenchymal transition via the TGF-β/Smad pathway in squamous cell carcinoma of the head and neck

**DOI:** 10.3892/or.2025.9001

**Published:** 2025-09-29

**Authors:** Changyun Yu, Yong Liu, Donghai Huang, Yaozhang Dai, Gengming Cai, Jinjie Sun, Ting Xu, Yongquan Tian, Xin Zhang

Oncol Rep 25: 1581–1587, 2011; DOI: 10.3892/or.2011.1251

Following the publication of the above paper, it was drawn to the Editor's attention by a concerned reader that, regarding the Transwell assay data shown in Fig. 2C and 4D, two pairs of overlapping sections of data were identified comparing the panels in these figures, where the results from differently performed experiments were intended to have been portrayed.

The authors were contacted by the Editorial Office to offer an explanation for this apparent duplication of data within the two figures; however, up to this time, no response from them has been forthcoming. Owing to the fact that the Editorial Office has been made aware of potential issues surrounding the scientific integrity of this paper, we are issuing an Expression of Concern to notify readers of this potential problem while the Editorial Office continues to investigate this matter further.

